# Both Natural and Induced Anti-Sda Antibodies Play Important Roles in GTKO Pig-to-Rhesus Monkey Xenotransplantation

**DOI:** 10.3389/fimmu.2022.849711

**Published:** 2022-03-29

**Authors:** Hao Feng, Tao Li, Jiaxiang Du, Qiangbing Xia, Lu Wang, Song Chen, Lan Zhu, Dengke Pan, Yi Wang, Gang Chen

**Affiliations:** ^1^ Institute of Organ Transplantation, Tongji Hospital, Tongji Medical College, Huazhong University of Science and Technology, Wuhan, China; ^2^ Key Laboratory of Organ Transplantation, Ministry of Education and National Health Commission (NHC), Chinese Academy of Medical Sciences, Wuhan, China; ^3^ Department of Organ Transplantation, The Transplantation Institute of Hainan Medical University, The Second Affiliated Hospital of Hainan Medical University, Hainan, China; ^4^ Genetic Engineering Department, Chengdu Clonorgan Biotechnology Co., Ltd, Chengdu, China; ^5^ Clinical Immunology Translational Medicine Key Laboratory of Sichuan Province, Academy of Medical Sciences and Sichuan Provincial People’s Hospital, Chengdu, China

**Keywords:** Sda, xenotransplantation, genetic engineering, pig, rhesus monkey

## Abstract

Sda, produced by the B4GALNT2 enzyme, has been recognized as an important xenoantigen for pig-to-nonhuman primate xenotransplantation. However, little is known about Sda expression in pigs and its immunogenicity in xenotransplantation. In this study, peripheral blood mononuclear cells (PBMCs) were isolated from wildtype, GTKO (with high, moderate, and low Sda expression), GTKO/β4GalNT2KO, GTKO/CMAHKO, or GTKO/CMAHKO/β4GalNT2KO pigs. Anti-pig IgM/IgG binding and complement-dependent cytotoxicity (CDC) to pig PBMCs was measured by flow cytometry using pooled rhesus monkey sera (n=20) or human sera (n=20). As compared to wild-type pigs (n=12), GTKO pigs (n=17) had a significantly higher mean level of Sda expression on PBMCs and showed a greater individual difference in expression. Both the overall binding of monkey serum IgM/IgG antibody to GTKO pig PBMCs and CDC against these PBMCs decreased significantly with a progressive reduction in Sda expression, showing a clear dose-effect relationship. Both the monkey serum antibody binding and CDC decreased significantly after the additional deletion of Sda, whereas the binding of human serum antibody and CDC against the GTKO pig PBMCs were markedly reduced after the deletion of Neu5Gc in the pigs. In addition, anti-Sda antibody accounted for > 50% of the induced anti-non-Gal antibody at the time of rejection in two rhesus monkeys that received GTKO/hCD55 pig kidney xenotransplantation, and the anti-Sda antibody showed significant cytotoxic activity against GTKO pig cells. We conclude that both natural and induced anti-Sda antibodies play important roles in GTKO pig-to-rhesus monkey xenotransplantation, thus providing further evidence for GTKO/β4GalNT2KO pigs as the preferred organ source for rhesus monkeys as a preclinical model of xenotransplantation.

## Introduction

Organ shortage remains a major problem for clinical organ transplantation worldwide (1). Xenotransplantation using genetically engineered pigs as organ donors is considered a promising way to solve this problem (2–4). To date, three carbohydrate xenoantigens expressed on porcine cells have been shown to play important roles in pig-to-human xenotransplantation: galactose-α1,3-galactose (Gal), N-glycolylneuraminic acid (Neu5Gc), and Sda. These xenoantigens can be deleted individually or in combination by using gene editing techniques (5–17). At present, a variety of genetically modified pigs have emerged, among which triple-knockout (TKO) pigs (GTKO/β4GalNT2KO/CMAHKO) are considered to be the best choice for future clinical trials (2–4, 11, 15, 16).

Preclinical studies using Old World nonhuman primates (NHP) as xenograft recipients had been standard practice for many years before pig-to-human xenotransplantation could become a reality. Baboons, rhesus monkeys, and cynomolgus monkeys have been the most commonly used Old World NHPs for preclinical xenotransplantation studies. Unlike humans, these Old World NHPs express Neu5Gc as pigs do, so they do not generate natural antibodies against Neu5Gc. Because of this difference, TKO pigs are not necessarily ideal donors for preclinical studies in the Old World NHPs. Several *in vitro* and *in vivo* studies have indicated that xenotransplantation into Old World monkeys or baboons using TKO pig donors is likely to be less effective than using GTKO/β4GalNT2KO pig donors, and even worse than using GTKO pig donors (11, 15–17). Andrew B. Adams’ group has reported that pre-transplant selection of recipients with low titers of anti-pig antibodies significantly prolongs renal xenograft survival in a GTKO/β4GalNT2KO pig-to-rhesus monkey kidney transplant model (12, 18). Therefore, GTKO/β4GalNT2KO pigs have been suggested as the preferred organ source for Old World NHPs as a preclinical model of xenotransplantation (17).

In recent years, the use of GTKO/β4GalNT2KO pigs has confirmed the presence of preformed anti-Sda antibodies in Old World NHPs and humans (12). However, although the Sda antigen has been known for many years, little is known about the expression and immunogenicity of the glycan produced by the porcine β4GalNT2 enzyme. In order to further clarify the important role of Sda in pig-to-rhesus monkey xenotransplantation, we have now designed *in vitro* experiments to investigate (i) the differences in Sda expression on peripheral blood mononuclear cells (PBMCs) in individual wildtype (WT) pigs and GTKO pigs as well as the two populations as a whole; (ii) the impact of different levels of Sda expression on monkey serum IgM/IgG antibody binding and complement-dependent cytotoxicity (CDC) against pig PBMCs with Gal deletion; (iii) potential differences in the role of Sda in pig-to-rhesus monkey vs. pig-to-human xenotransplantation; and (iv) whether the expression of Sda can induce a significant antibody response after GTKO pig-to-rhesus monkey xenotransplantation and thus play an important role in the development of acute humoral xenograft rejection.

## Materials and Methods

### Sources of Rhesus Monkey and Human Sera

Monkey sera were obtained from 20 outbred male rhesus monkeys (4-10 years old, Hubei Tianqin Biotechnology Corporation and South China Primates Research Center, Hubei, China). Human sera were obtained from 20 healthy human volunteers (22–44 years old, both genders) who had no history suggesting previous exposure to pig antigens or to alloantigens (such as blood transfusions, a previous failed renal allograft, or pregnancy). Monkey or human sera were stored in as either single or pooled samples at −80°C. When required, decomplementation was carried out by heat-inactivation for 30 min at 56°C.

We had previously performed GTKO pig-to-rhesus monkey kidney xenotransplants in another study; two of the recipients had developed acute humoral xenograft rejection and graft failure at 19 days post-transplant. The recipients’ sera collected 6 days before transplantation and at the time of rejection (day 19) were stored at -80°C and were used in the present study.

### Sources of Pig Cells

**Figure 1:**
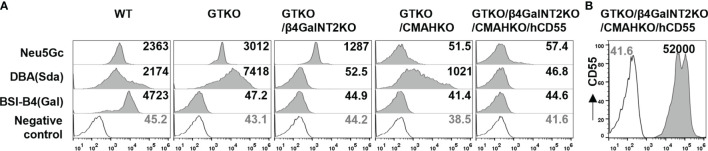
Expression of Gal, Sda, and Neu5Gc on pig PBMCs. **(A)** The expression of Gal, Sda, and Neu5Gc on pig PBMCs from wildtype (WT), GTKO, GTKO/β4GalNT2KO, GTKO/CMAHKO, and TKO/hCD55 pigs (Gmean values were shown). White histograms outlined in black represent negative controls that were unstained cells for lectin experiments. **(B)** The expression of human CD55 (hCD55) on TKO/hCD55 pig PBMCs.

PBMCs were obtained from WT (n=12); α1,3-galactosyltransferase gene knockout (GTKO) (n=17); α1,3-galactosyltransferase/β1,4-N-acetyl-galactosaminyl transferase 2 gene double-knockout (GTKO/β4GalNT2KO) (n=1); α1,3-galactosyltransferase/CMP-N-acetylneuraminic acid hydroxylase gene double-knockout (GTKO/CMAHKO); (n=1) or α1,3-galactosyltransferase/β1,4-N-acetyl-galactosaminyl transferase 2/CMP-N-acetylneuraminic acid hydroxylase gene triple-knockout and human CD55 transgenic (GTKO/β4GalNT2KO/CMAHKO/hCD55; TKO/hCD55, n=1) pigs (Chengdu Clonorgan Biotechnology Co., LTD, Chengdu, China), all of blood type O and the same genetic background (Bama miniature pig). The pig PBMCs from the various donors were isolated by Ficoll density gradient centrifugation as described (19). Representative phenotypic identification results are shown in **Figure 1**. To study the role of Sda expression in pig-to-rhesus monkey xenotransplantation, two wild-type pigs with a high (WT-Sda^hi^) or low (WT-Sda^lo^) level of Sda expression and three GTKO pigs with high (GTKO-Sda^hi^), moderate (GTKO-Sda^mo^) or low (GTKO-Sda^lo^) Sda expression were selected to provide PBMCs for related *in vitro* experiments. Although the expression of human complement regulatory protein in transgenic pigs would affect the complement-dependent cytotoxicity (CDC) results, because we were unable to obtain a pure GTKO/β4GalNT2KO/CMAHKO (TKO) pig, we used a TKO/hCD55 pig instead.

### Detection of the Expression of Gal, Sda, Neu5Gc, and Human CD55 on Porcine PBMCs by Flow Cytometry

PBMCs were isolated from the peripheral blood of each pig, and 100 μl of cell suspension containing 5×10^5^ PBMCs were dispensed into each FACS tube. Pig PBMCs were stained for the expression of Gal (by isolectin BSI-B4, 1:500, Sigma-Aldrich, St. Louis, MO), Sda (with Dolichos biflorus agglutinin, DBA, 1:1000, Vector Labs, Burlingame, CA, USA), and Neu5Gc (with chicken anti-Neu5Gc mAb, 1:800, Biolegend, San Diego, CA). The expression of human CD55 on PBMCs from the TKO/hCD55 pig was detected by using allophycocyanin (APC)-conjugated anti-human CD55 (1:100, Biolegend, San Diego, CA). Samples were analyzed on a flow cytometer (Cytoflex S, Beckman Coulter, USA), and the data were analyzed using FlowJo software (Treestar, San Carlos, CA). The results are reported as geometric mean fluorescence (Gmean).

### Detection of the Binding of Serum IgM and IgG to Pig PBMCs by Flow Cytometry

Serum IgM and IgG binding to pig PBMCs were measured by flow cytometry, using a technique similar to that previously described (19, 20). In brief, 100 μl of isolated pig PBMCs (5 × 10^5^ cells/tube) were incubated with 100 µl diluted rhesus monkey or human serum (10% final concentration, heat-inactivated for 30 min at 56°C in advance) for 30 min at 4°C. Cells incubated with 100 µl of phosphate-buffered saline (PBS) served as negative controls. After incubation, the cells were washed twice in PBS and centrifuged at 400 g for 5 min. The supernatant was discarded. To prevent non-specific binding, 200 µl of 10% heat-inactivated goat serum (Solarbio, Beijing, China) was added, and incubation was performed for 15 min in the dark at 4°C. After a wash with FACS buffer, the cells were incubated for 30 min with donkey anti-human IgM (1:1600) or goat anti-human IgG (1:1600) conjugated to Alexa-488 (Jackson ImmunoResearch Laboratories Inc., West Grove, PA) or APC-conjugated mouse anti-human IgG (1:1000, Biolegend, San Diego, CA). After incubation, the samples were washed twice and then resuspended with 200 µl PBS. The stained cells were analyzed by flow cytometry (Cytoflex S, Beckman Coulter, USA), and the data were analyzed using FlowJo software (Treestar, San Carlos, CA). The results were expressed as the Gmean. Each sample was repeatedly measured in three independent experiments.

### Complement-Dependent Cytotoxicity (CDC) Assay for PBMCs

CDC assays were performed and monitored by flow cytometry using WT-Sda^hi^, WT-Sda^lo^, GTKO-Sda^hi^, GTKO-Sda^mo^, GTKO-Sda^lo^, GTKO/β4GalNT2KO, GTKO/CMAHKO, or TKO/hCD55 pig PBMCs as target cells, as described in detail (19). In brief, pig PBMCs (5 × 10^5^ cells in 100 µl FACS buffer) were incubated with 100 µl of diluted serum for 1 h at 4°C. The final serum concentration varied from 50% to 1.57%. After a wash with FACS buffer, 50 µl of rabbit complement (Cedarlane, Hornby, CA, USA) were added to each tube and incubated for 30 min at 37°C. After the incubation, the cells were washed with FACS buffer, and then 100 µl of propidium iodide (2 µg/ml, eBioscience, Inc., CA, USA) were added to each tube. After further incubation in the dark for 15 min at 4°C, the cells were examined by flow cytometry (Cytoflex S, Beckman Coulter, USA). The results were analyzed using FlowJo software, and the percentage of propidium iodide-positive cells was used to determine the extent of the CDC that occurred.

### Statistical Analysis

Data are presented as means ± SD. The variation of individuals within each group was indicated by coefficient of variation (C.V=SD/mean x 100%). The significance of the difference between two groups was determined by unpaired *t*-test. Comparisons among multiple groups were performed using one-way analysis of variance (ANOVA) with Tukey’s multiple comparison test. All statistical analyses were performed using Graph Pad Prism version 7 software (GraphPad Software, USA). *P <*0.05 was considered statistically significant.

## Results

### Expression of Gal, Sda, and Neu5Gc on PBMCs From Different Types of Knockout Pigs

We first assessed the expression of three carbohydrate xenoantigens (Gal, Sda, and Neu5Gc) in pigs with different types of gene knockout. FACS analysis revealed that 1) PBMCs from wild-type pigs expressed all three xenoantigens; 2) GTKO pig PBMCs expressed Sda and Neu5Gc, but not Gal; 3) PBMCs from GTKO/β4GalNT2KO pigs did not express Gal or Sda; 4) PBMCs from GTKO/CMAHKO pigs did not express Gal or Neu5Gc; and 5) TKO/hCD55 pig PBMCs did not express any of the three xenoantigens (**Figure 1**).

### The Expression of Sda in WT or GTKO Pig PBMCs

**Figure 2:**
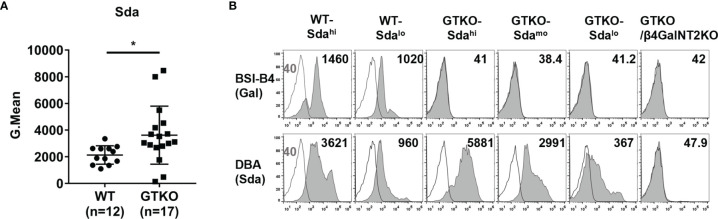
The expression of Sda in WT or GTKO pigs. PBMCs from WT, GTKO, or GTKO/β4GalNT2KO pigs were stained for the expression of Gal (by isolectin BSI-B4) and Sda (with Dolichos biflorus agglutinin, DBA). The results are reported as geometric mean fluorescence (Gmean). **(A)** Comparison of the expression of Sda on WT (n=12) and GTKO (n=17) pig PBMCs. Data are presented as means ± SD (*P<0.05); **(B)** The expression of Gal and Sda on PBMCs from two wild-type pigs and three GTKO pigs with different levels of Sda expression. White histograms outlined in black represent negative controls that were unstained cells for lectin experiments. The appearance of a single histogram in GTKO or GTKO/β4GalNT2KO samples indicates an overlap with the negative control.

To determine whether there was a difference in Sda expression between wild-type and GTKO pigs and whether there were large individual differences within each population, we performed FACS assays to detect Sda expression on PBMCs from 12 wild-type pigs and 17 GTKO pigs. As shown in **Figure 2A**, there was a relatively low individual variation in the expression of Sda in wild-type pigs (Gmean: 1094-3345, SD=690, C.V=32.4%), but the individual variation in Sda expression in GTKO pigs was much greater (Gmean: 140-8465, SD=2175, C.V=60.1%). As compared to wild-type pigs, GTKO pigs had a higher mean level of Sda expression on their PBMCs (Gmean: 3618 ± 2175 vs., 2130 ± 690, *p*=0.03).

To study the role of Sda expression in pig-to-rhesus monkey xenotransplantation, we selected two wild-type pigs and three GTKO pigs with different levels of Sda expression to provide PBMCs for our subsequent *in vitro* experiments. The expression of Sda on the PBMCs from the two wild-type pigs was high (WT-Sda^hi^) in one case and low (WT-Sda^lo^) in the other, and the expression of Sda on the PBMCs of the three GTKO pigs was high (GTKO-Sda^hi^) in one case, moderate (GTKO-Sda^mo^) in the second, and low (GTKO-Sda^lo^) in the third (**Figure 2B**).

### Natural Anti-Pig Antibody Levels in Serum of Rhesus Monkeys Show Great Individual Differences

**Figure 3:**
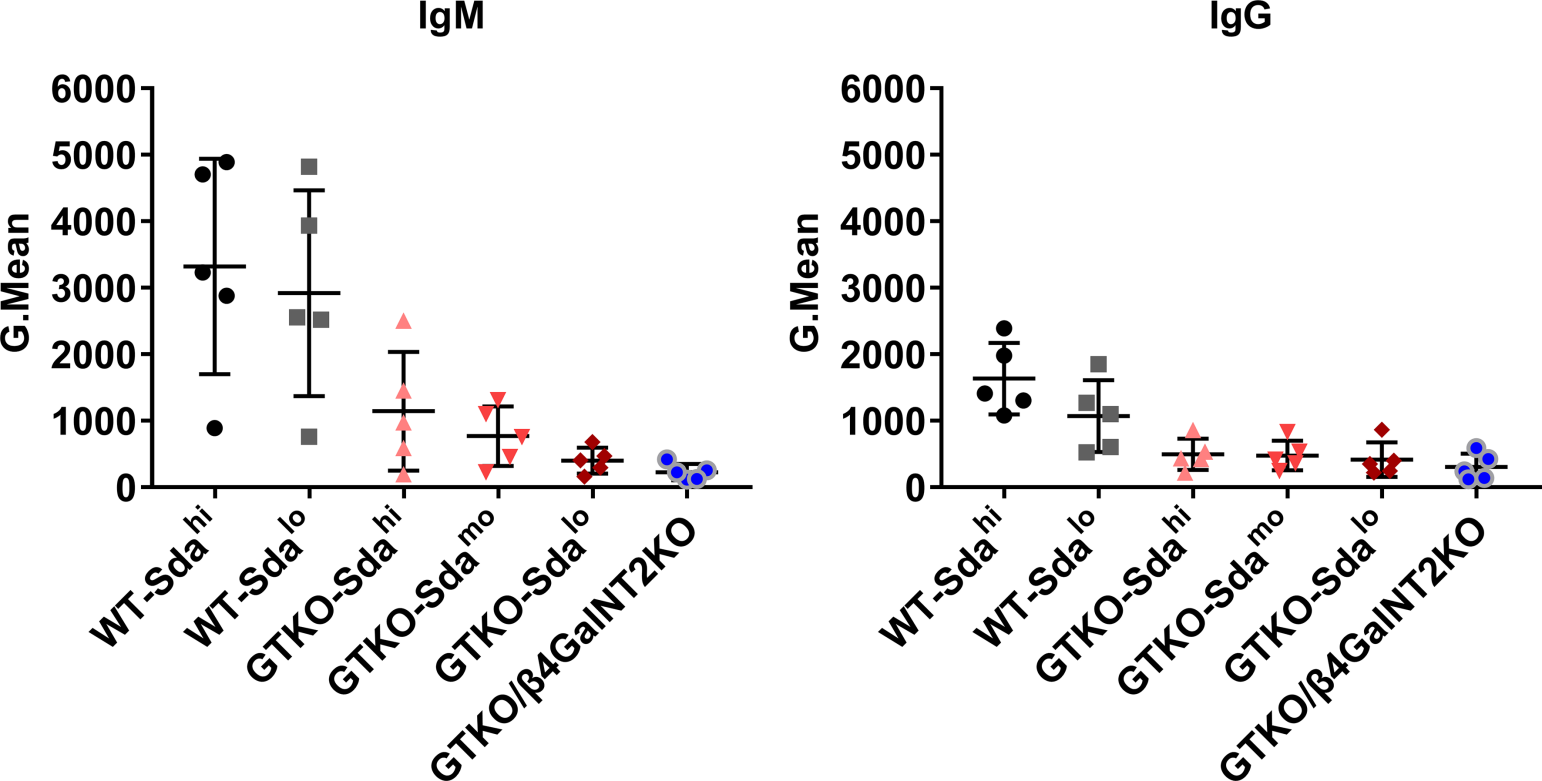
Natural anti-pig antibody levels in the serum of rhesus monkeys. Five of 20 rhesus monkeys that had not been immunologically screened were randomly selected. Both anti-pig IgM and IgG levels in the serum (diluted at 1:10) of each monkey were measured by flow cytometry using PMBCs from WT (Sda^hi^ and Sda^lo^), GTKO (Sda^hi^, Sda^mo^, and Sda^lo^) or GTKO/β4GalNT2KO pigs as the source of the target cells. The results are reported as Gmean. Data are presented as means ± SD.

To determine whether there are large individual differences in the levels of preformed anti-Gal and anti-Sda antibodies in rhesus monkeys, we randomly selected 5 of 20 rhesus monkeys that had not been immunologically screened, and measured both anti-pig IgM and IgG levels in the serum of each monkey by flow cytometry using PMBCs from WT (Sda^hi^ and Sda^lo^), GTKO (Sda^hi^, Sda^mo^, and Sda^lo^) or GTKO/β4GalNT2KO pigs as the source of the target cells. When PBMCs from WT pigs were used as the target cells for antibody detection, the levels of both anti-pig IgM and IgG in serum of the five monkeys showed great individual differences, regardless of the high or low expression of Sda on the porcine PBMCs (SD values of the WT-Sda^hi^ and WT-Sda^lo^ groups were 1620 and 1550 (IgM), 537.3 and 538.6 (IgG), respectively), suggesting that there were large individual differences in the levels of anti-Gal antibodies (**Figure 3**). When PBMCs from GTKO pigs were used as the target cells for antibody detection, the results reflected the level of antibodies against non-Gal antigens. As shown in **Figure 3**, the anti-non-Gal IgM levels showed an increasingly smaller individual difference as the Sda expression decreased on the porcine PBMCs, whereas the individual differences in the IgG levels did not show a similar pattern (from left to right, SD values of IgM were 892.7, 447.1, 194.7, and 122.8; SD values of IgG were 234.9, 223.6, 261.9, and 200.4). These results suggest that, at the least, the levels of serum anti-Sda IgM antibodies vary greatly among different rhesus monkeys. In order to avoid interference caused by these individual differences, we mixed serum samples from 20 monkeys together for subsequent studies.

### Sda Is the Major Xenoantigen for the Binding of Monkey Serum Antibody to GTKO Pig PBMCs and Plays an Important Role in CDC Against These Cells

**Figure 4:**
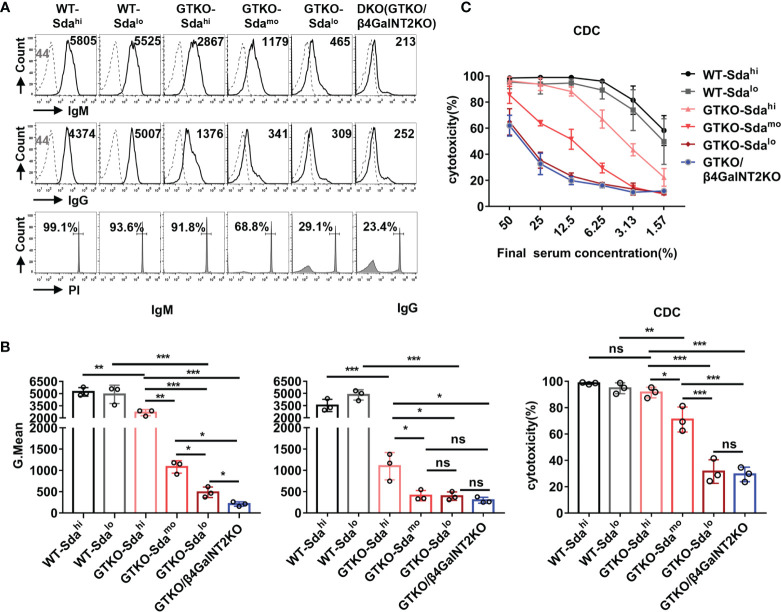
The role of Sda expression in the binding of monkey serum antibody to GTKO pig PBMCs and in CDC against these cells. The IgM/IgG antibody binding and CDC were measured by flow cytometry after the co-culture of the pooled monkey sera with PMBCs from wildtype (Sda^hi^ and Sda^lo^), GTKO (Sda^hi^, Sda^mo^, and Sda^lo^), or GTKO/β4GalNT2KO pigs. **(A)** Flow cytometric histograms showing representative binding of rhesus monkey IgM/IgG antibody (top/middle row, serum diluted 1:10, Gmean values were shown) to pig PBMCs and CDC (bottom row, serum diluted 1:6, percentages were shown) against the same PBMCs. **(B)** Statistical analysis of pooled rhesus monkey serum antibody binding (IgM and IgG) to pig PBMCs and CDC against the same PBMCs. **(C)** Comparison of mean serum CDC (at serum concentrations of 50% to 1.57%) of pooled rhesus monkey against WT, GTKO, or GTKO/β4GalNT2KO pig PBMCs. Each sample was measured three times. All data are presented as means ± SD (*P<0.05, **P< 0.01, ***P< 0.001; ns, non-significant). The P value comes from three independent experiments.

The IgM/IgG antibody binding and CDC were measured by flow cytometry after the co-culture of the pooled monkey sera with PMBCs from wildtype (Sda^hi^ and Sda^lo^), GTKO (Sda^hi^, Sda^mo^, and Sda^lo^), or GTKO/β4GalNT2KO pigs. As shown in **Figure 4A**, both the antibody binding (IgM and IgG) and CDC against WT pig PBMCs were very high (GMean of IgM: ~5000; GMean of IgG: ~4000; CDC: >90%), regardless of the high or low expression of Sda on the porcine PBMCs. In contrast, the binding of serum IgM and IgG antibody to PBMCs from GTKO pigs was significantly lower than that to WT pig PBMCs (*P*<0.01). These results confirm that Gal is still the most important xenoantigen in pig-to-monkey xenotransplantation.

Notably, the monkey serum IgM/IgG binding to GTKO pig PBMCs (Gal-negative) with high expression of Sda remained relatively high (GMean of IgM: ~2700; GMean of IgG: ~1000), a level of reactivity that could still mediate a high level of CDC (>90%) at a serum concentration from 12.5% to 50%. When compared to the results obtained using GTKO pig PBMCs with different levels of Sda expression (high, moderate, low, negative) as target cells, the overall monkey serum IgM/IgG antibody binding and CDC to pig PBMCs decreased significantly with the reduction in Sda expression (**Figure 4B**). This result was confirmed by using PBMCs from another group of GTKO pigs with high, medium and low Sda expression (**Supplemental Figure 1**). The level of CDC against GTKO pig PBMCs with low expression of Sda was almost the same as that to GTKO/β4GalNT2KO pig PBMCs (*P*>0.05) (**Figures 4B, C**). These *in vitro* results suggest that the monkey serum-mediated CDC against GTKO pig PBMCs is closely related to the expression level of Sda; thus, Sda may play a major role in GTKO pig-to-rhesus monkey xenotransplantation.

### In the Absence of the Gal Antigen, Human Antibody Binding to Porcine PBMCs Is Dependent on the Presence of Neu5Gc, Whereas Monkey Antibody Binding Depends on Sda

**Figure 5:**
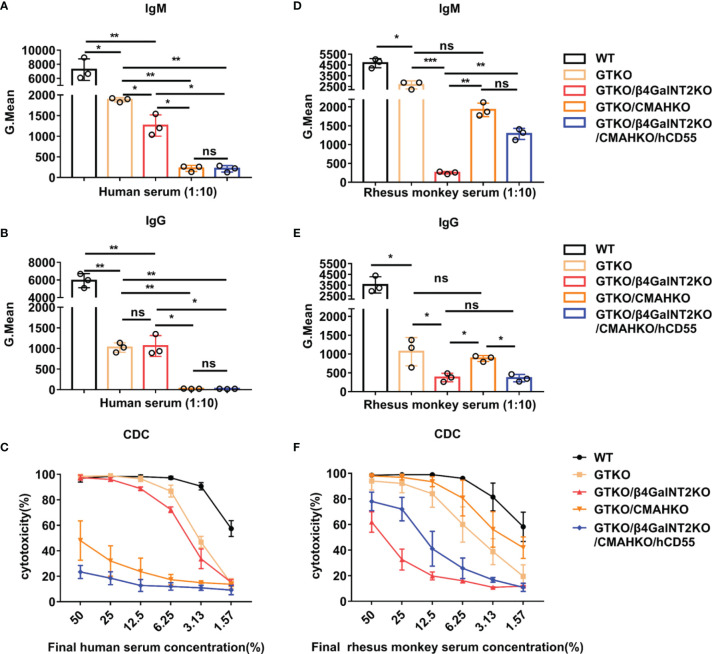
The role of Sda and Neu5Gc expression in the binding of human vs. monkey serum antibody to GTKO pig PBMCs and in CDC against these cells. IgM/IgG antibody binding and CDC were measured by flow cytometry after co-culture of pooled human or monkey sera with PMBCs from WT, GTKO, GTKO/β4GalNT2KO, GTKO/CMAHKO, or TKO/hCD55 pigs. **(A, B, D, E)** Pooled human or rhesus monkey serum IgM/IgG binding (at a serum concentration of 10%) to pig PBMCs. **(C, F)** Pooled human or rhesus monkey serum CDC (at serum concentrations of 50% to 1.57%) against pig PBMCs. Each sample was measured three times. All data are presented as means ± SD (*P<0.05, **P< 0.01, ***P< 0.001; ns, non-significant). The P value comes from three independent experiments.

Next, we investigated the importance of various porcine xenoantigens in transplantation into humans and rhesus monkeys. For this purpose, IgM/IgG antibody binding and CDC were measured by flow cytometry after co-culture of pooled human or monkey sera with PMBCs from WT, GTKO, GTKO/β4GalNT2KO, GTKO/CMAHKO, or TKO/hCD55 pigs. In the case of both humans and rhesus monkeys, the binding of serum IgM and IgG to GTKO pig PBMCs (Gal deletion) was significantly lower than that to wild-type pig PBMCs (*P*<0.01) (**Figures 5A, B, D, E**). The binding of human serum IgM to GTKO/β4GalNT2KO pig PBMCs (both Gal and Sda deletion) was slightly lower than that to GTKO pig PBMCs (*P*<0.05), but there was no significant change in serum IgG binding (*P*>0.05). In contrast, both human serum IgM and IgG binding to GTKO/CMAHKO pig PBMCs (both Gal and Neu5Gc deletion) was dramatically lower than that to GTKO pig PBMCs (*P*<0.01) and almost close to the binding levels to TKO/hCD55 pig PBMCs (Gal, Sda, and Neu5Gc deletion) (**Figures 5A, B**). In addition, the cytotoxicity of human serum against porcine PBMCs decreased only at lower serum concentrations (6.25%-1.57%) after the deletion of Gal alone or both Gal and Sda in pigs, whereas the cytotoxicity of human serum against porcine PBMCs was markedly decreased at all serum dilutions after the deletion of Gal and Neu5Gc in pigs (**Figure 5C**). These results suggest that next to anti-Gal antibody, anti-Neu5Gc antibody is the main preformed anti-pig antibody in human serum.

Unlike the results we obtained for pooled human serum, the binding of rhesus monkey serum IgM antibody to GTKO/β4GalNT2KO pig PBMCs was significantly lower than that to pig PBMCs from WT, GTKO, GTKO/CMAHKO, or even TKO/hCD55 pigs (*P*< 0.01 or 0.001) (**Figure 5D**). The binding of monkey serum IgG antibody to GTKO/β4GalNT2KO pig PBMCs was also significantly lower than that to pig PBMCs from WT, GTKO, or GTKO/CMAHKO pigs (*P*<0.05 or 0.01) but similar to the binding to TKO/hCD55 pig cells (**Figure 5E**). Consistent with the antibody binding results, the cytotoxicity of monkey serum against GTKO/β4GalNT2KO pig PBMCs was significantly lower than that against WT, GTKO, GTKO/CMAHKO, or even TKO/hCD55 pig PBMCs (**Figure 5F**). These results indicate that Sda is more important for pig-to-rhesus monkey xenotransplantation, and GTKO/β4GalNT2KO pigs are a more suitable organ source than the other pigs for rhesus monkeys as a preclinical model of xenotransplantation.

### Induced Antibodies Against Sda Play an Important Role in the Development of Acute Humoral Xenograft Rejection in GTKO/hCD55 Pig-to-Rhesus Monkey Kidney Xenotransplantation

We have performed four renal xenotransplants in rhesus monkeys using kidneys from GTKO/hCD55 pigs. The recipient monkeys received induction therapy with anti-thymocyte globulin (thymoglobulin) and anti-CD20mAb (rituximab) and maintenance therapy with tacrolimus, mycophenolate mofetil, and low-dose corticosteroids. Two of the four recipient monkeys (R01 and R02) generated high levels of circulating antibodies against non-Gal antigens and developed acute humoral xenograft rejection on day 19 (data not shown). In order to study the role of anti-Sda antibody in xenograft rejection, we used serum samples collected before transplantation (day -6) and at the time of rejection (day 19) to measure IgM/IgG antibody binding and CDC against GTKO or GTKO/β4GalNT2KO pig PBMCs.

**Figure 6:**
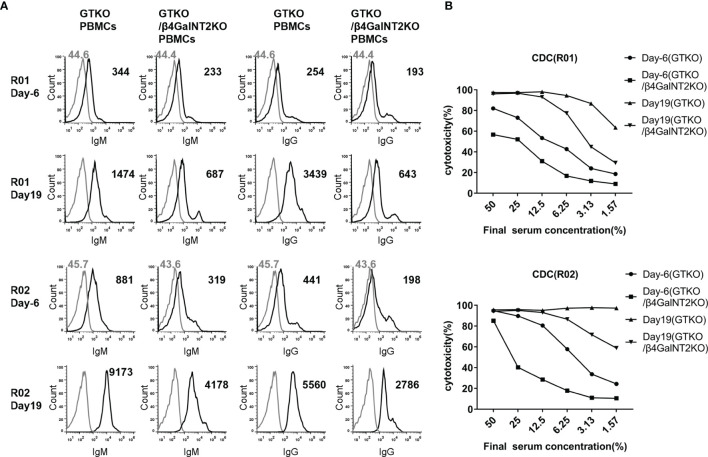
The role of induced antibodies against Sda in the development of acute humoral xenograft rejection in GTKO/hCD55 pig-to-rhesus monkey kidney xenotransplantation. Renal xenotransplantation was previously performed in four rhesus monkeys using kidneys from GTKO/hCD55 pigs. Two of the four recipient monkeys (R01 and R02) generated high levels of circulating antibodies against non-Gal antigens and developed acute humoral xenograft rejection on day 19. Serum samples collected before transplantation (day -6) and at the time of rejection (day 19) were used to measure IgM/IgG antibody binding and CDC against GTKO (Sda^mo^) or GTKO/β4GalNT2KO pig PBMCs. **(A)** The FACS results of serum IgM and IgG binding to pig PBMCs (at a serum concentration of 10%). Cells were incubated with medium served as negative controls (gray lines). The number represents the geometric mean fluorescence intensity (Gmean). **(B)** Comparison of CDC (at serum concentrations of 50% to 1.57%) of rhesus monkey (R01 and R02) pre-transplant (day -6) and post-transplant (day 19) serum against GTKO and GTKO/β4GalNT2KO pig PBMCs.

As shown in **Figure 6**, the binding of IgM and IgG antibodies to GTKO pig PBMCs was significantly (4 to 14 times) higher in the serum of both monkeys on post-transplant day 19 than in serum collected before transplantation (day -6), indicating that a large proportion of the antibody against non-Gal antigens was newly generated after transplantation. To analyze whether these anti-non-Gal antibodies contain a significant amount of anti-Sda antibodies, we compared the results of antibody binding assays using GTKO pig PBMCs (Sda^mo^) and GTKO/β4GalNT2KO pig PBMCs as target cells. In the sera of both monkeys at 19 days after transplantation, the levels of IgM and IgG binding to GTKO/β4GalNT2KO pig PBMCs were significantly lower than those to GTKO pig PBMCs (IgM decreased by 53% and 54%, IgG decreased by 81% and 50%, respectively), suggesting that anti-Sda antibodies accounted for more than half of the non-Gal-specific antibodies generated after transplantation (**Figure 6A**). In addition, we measured CDC against pig PBMCs using the four serum samples from the two monkeys before and after transplantation, and the results showed that the cytotoxicity of the same serum to GTKO/β4GalNT2KO pig PBMCs was lower than that against GTKO pig PBMCs, indicating that anti-Sda antibodies have cytotoxic activity against pig cells (**Figure 6B**).

## Discussion

Rhesus monkeys are often used in pig-to-NHP xenotransplantation studies (6, 12, 21, 22). There may be differences in natural anti-pig antibodies and immune reactivity to pig xenoantigens between rhesus monkeys and humans, so the genetically engineered pigs that favor long-term xenograft survival in rhesus monkeys may differ from those that are suitable for humans. In the present study, we clearly demonstrate through a series of *in vitro* experiments that, after Gal, Sda is the most important xenoantigen for pig-to-rhesus monkey xenotransplantation, whereas Neu5Gc is more important for pig-to-human xenotransplantation. In addition, we provide the first evidence that Sda can induce a significant antibody response in GTKO/hCD55 pig-to-rhesus monkey kidney xenotransplantation, and also that induced anti-Sda antibodies play an important role in the development of acute humoral xenograft rejection. These results suggest that a GTKO/β4GalNT2KO pig will be more advantageous than the other strains tested for a pig-to-rhesus monkey xenotransplant model.

Most pig-to-NHP xenotransplantation studies to date have used GTKO pigs with or without the additional expression of certain human transgenic proteins (6, 16, 21–24). In these studies, animals with low levels of anti-pig antibodies and CDC were often selected as recipients for better graft survival (21, 22). However, few studies have measured the expression levels of xenoantigens other than Gal in donor pigs. Sda may be the most important xenoantigen for NHPs after Gal. In the present study, we have shown for the first time (to our knowledge) that the mean level of Sda expression in GTKO pigs is significantly higher than that in WT pigs, which may be related to the increased compensatory expression of other carbohydrate xenoantigens after Gal knockout. In addition, we found great differences in Sda expression level among individual GTKO pigs. These novel findings suggest that it is also necessary to assess Sda expression in donor pigs before GTKO pig-to-NHP xenotransplantation.

In 2015, Estrada JL et al. conducted *in vitro* studies using PBMCs from GTKO, GTKO/CMAHKO, and TKO pigs and found that inactivation of the β4GalNT2 gene reduces the binding of human and NHP antibody to pig cells (11). These results suggested that Sda expression in pigs may play a role in xenograft rejection. However, because of the lack of GTKO/β4GalNT2KO pigs in that study, the specific role of Sda antigen in xenotransplantation remains uncertain. In the last 3 years, two studies used rhesus monkey serum (n=14) and baboon serum (n=72), respectively, to perform *in vitro* antibody binding assays and CDC assays using PBMCs from GTKO, GTKO/β4GalNT2KO, or TKO pigs and found that the binding of both rhesus and baboon serum antibody and CDC against GTKO/β4GalNT2KO pig cells were significantly reduced (12, 17). In the current study, we have used GTKO pigs with different levels of Sda expression (high, moderate, low) and GTKO/β4GalNT2KO pigs to provide PBMCs and found that the overall binding of monkey serum IgM/IgG antibody and CDC against pig PBMCs decreased significantly as Sda expression was reduced. The clear dose-effect relationship that we observed confirms the important role of Sda in GTKO pig-to-rhesus monkey xenotransplantation. In addition, we found that the levels of monkey serum antibody binding and CDC against GTKO pig PBMCs with a low expression of Sda were almost the same as those to GTKO/β4GalNT2KO pig PBMCs, suggesting that selecting GTKO pigs with low Sda expression as donors may achieve effects similar to those achieved by using GTKO/β4GalNT2KO pigs in a pig-to-rhesus xenotransplantation model.

We used pooled rhesus monkey sera and pooled human sera to perform *in vitro* antibody binding and CDC experiments with PBMCs from WT, GTKO, GTKO/B4GalNT2KO, GTKO/CMAHKO, and TKO pigs and demonstrated that Neu5Gc is important for pig-to-human xenotransplantation, whereas Sda is more important for pig-to-rhesus monkey xenotransplantation. These results are consistent with previous reports (11, 12, 15, 17) and indicate that preclinical xenotransplantation studies using NHPs do not fully mimic the immune response in humans after xenotransplantation. When conducting preclinical studies with rhesus monkeys or baboons, we need to consider using genetically edited pigs that are different from those suitable for humans.

Thus far, it has remained unclear whether the expression of Sda can induce a significant antibody response after GTKO pig-to-rhesus monkey xenotransplantation and subsequently contribute to the development of acute humoral xenograft rejection. Only one published paper has reported that there is positive antibody deposition in the rejected renal xenografts of long-term survivors and positive staining for the Sda antigen, suggesting that some of the late antibody-mediated injury may be directed against Sda (22). In the present study, we collected serum samples from two monkeys who had received GTKO pig kidney transplantation, which generated high levels of circulating non-Gal antibodies and developed acute humoral xenograft rejection. By measuring IgM/IgG antibody binding and CDC against GTKO or GTKO/β4GalNT2KO pig PBMCs, we found that anti-Sda antibodies accounted for more than half of the induced anti-non-Gal antibodies at the time of rejection and that anti-Sda antibodies showed significant cytotoxic activity against pig cells. To our knowledge, this is the first demonstration that the Sda antigen can induce significant antibody production and play an important role in mediating xenograft rejection in a pig-to-rhesus model. Since *in vivo* antibody binding is expected to occur first on endothelial cells of the graft in xenotransplantation, it is worth further attention whether the antibody binding results detected by donor PBMCs *in vitro* can reflect the “true danger” of the xenograft in the recipient.

In conclusion, we have demonstrated, for the first time that induced anti-Sda antibodies as well as natural anti-Sda antibodies play an important role in GTKO pig-to-rhesus monkey xenotransplantation, providing further evidence for GTKO/β4GalNT2KO pigs as the preferred organ source for rhesus monkeys used as recipients in a preclinical model of xenotransplantation.

## Data Availability Statement

The raw data supporting the conclusions of this article will be made available by the authors, without undue reservation.

## Ethics Statement

The studies involving human participants were reviewed and approved by ethics committee of Tongji Hospital, Tongji Medicine College, Huazhong University of Science and Technology (TJ-C20181002). All human subjects were adults. Written informed consent for participation was not required for this study in accordance with the national legislation and the institutional requirements. The animal study was reviewed and approved by Institutional Animal Care and Use Committee (IACUC) at the Tongji Hospital, Tongji Medical College, Huazhong University of Science and Technology (TJH-201905007).

## Author Contributions

GC and YW designed the experiments. HF, TL, JD, QX, and LW performed the *in vitro* experiments. GC, SC, and LZ performed the transplant surgery. DP and JD provided different genetically engineered pigs. HF, TL, JD, and GC analyzed the data and prepared the figures. HF wrote the article. GC critically revised the article. All authors contributed to the article and approved the submitted version.

## Funding

This study was supported by the Major Scientific and Technological Project of Hainan province (ZDKJ2019009). The funder was not involved in the study design, collection, analysis, interpretation of data, the writing of this article or the decision to submit it for publication.

## Conflict of Interest

Author JD is employed by Chengdu Clonorgan Biotechnology Co., LTD.

The remaining authors declare that the research was conducted in the absence of any commercial or financial relationships that could be construed as a potential conflict of interest.

## Publisher’s Note

All claims expressed in this article are solely those of the authors and do not necessarily represent those of their affiliated organizations, or those of the publisher, the editors and the reviewers. Any product that may be evaluated in this article, or claim that may be made by its manufacturer, is not guaranteed or endorsed by the publisher.

## Supplementary Material

Supplementary Figure 1 The role of Sda expression in the binding of monkey serum antibody to PBMCs from another group of GTKO pigs and in CDC against these cells. The IgM/IgG antibody binding and CDC were measured by flow cytometry after the co-culture of the pooled monkey sera with PMBCs from another group of GTKO pigs (Sda^hi^, Sda^mo^, and Sda^lo^). **(A)** The expression of Gal and Sda on PBMCs from three GTKO pigs with different levels of Sda expression. **(B)** Flow cytometric histograms showing binding of rhesus monkey IgM/IgG antibody (top/middle row, serum diluted 1:10, Gmean values were shown) to pig PBMCs and CDC (bottom row, serum diluted 1:6, percentages were shown) against the same PBMCs.

## Abbreviations

APC, allophycocyanin; CDC, complement-dependent cytotoxicity; CMAH, CMP-N-acetylneuraminic acid hydroxylase; DBA, Dolichos biflorus agglutinin; GTKO, α1,3-galactosyltransferase gene knockout; NHP, nonhuman primate; PBMCs, peripheral blood mononuclear cells; TKO, triple-knockout; β4GalNT2, β1,4-N-acetyl-galactosaminyl transferase 2.
