# Supplementary figures and images for: Both Natural and Induced Anti-Sda Antibodies Play Important Roles in GTKO Pig-to-Rhesus Monkey Xenotransplantation
Source: Front Immunol. 2022 Mar 29;13:849711. doi: 10.3389/fimmu.2022.849711 (PMC9004458; doi:10.3389/fimmu.2022.849711)

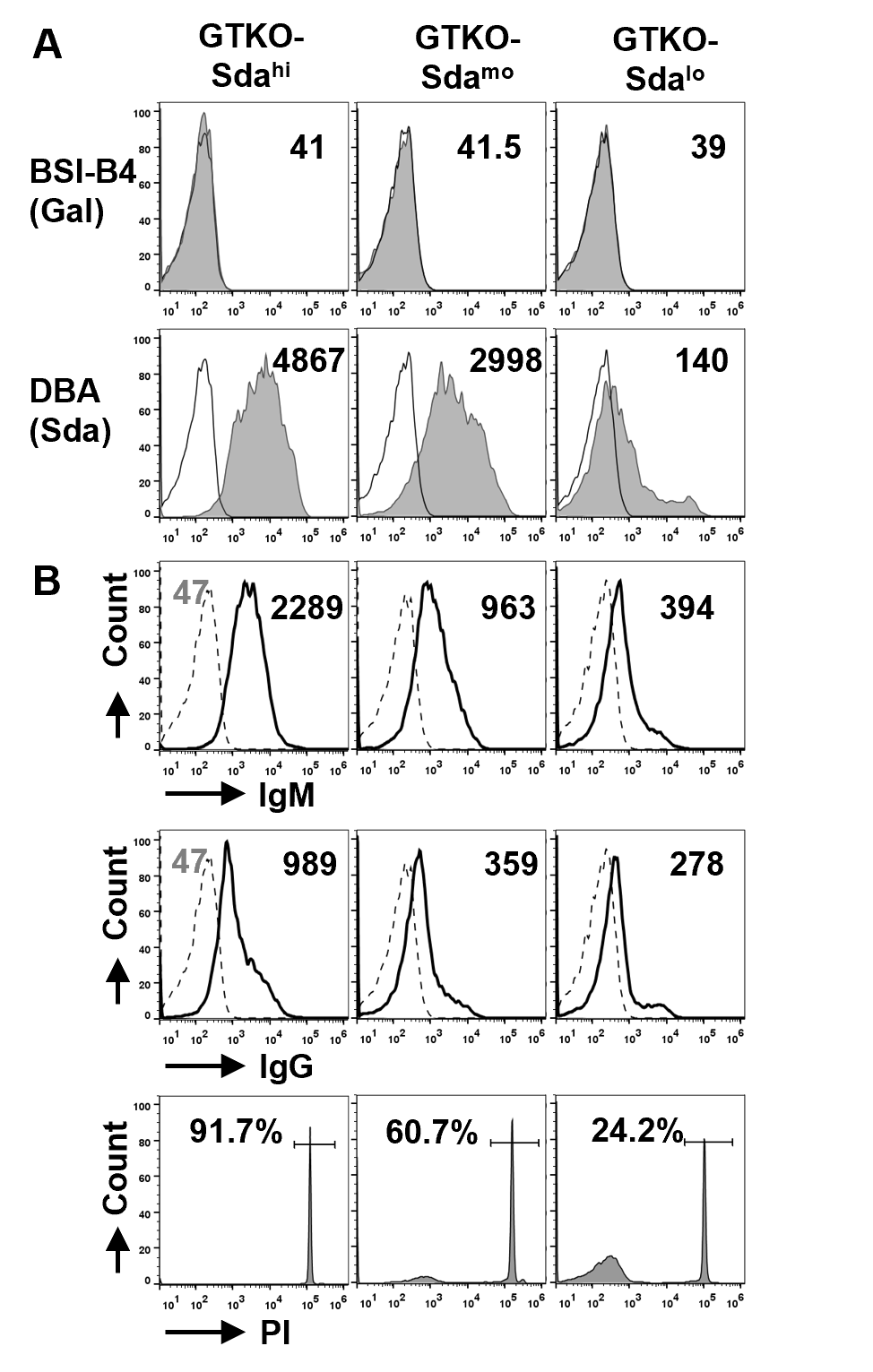

Supplement: Supplementary Figure 1 — The role of Sda expression in the binding of monkey serum antibody to PBMCs from another group of GTKO pigs and in CDC against these cells. The IgM/IgG antibody binding and CDC were measured by flow cytometry after the co-culture of the pooled monkey sera with PMBCs from another group of GTKO pigs (Sdahi, Sdamo, and Sdalo). (A) The expression of Gal and Sda on PBMCs from three GTKO pigs with different levels of Sda expression. (B) Flow cytometric histograms showing binding of rhesus monkey IgM/IgG antibody (top/middle row, serum diluted 1:10, Gmean values were shown) to pig PBMCs and CDC (bottom row, serum diluted 1:6, percentages were shown) against the same PBMCs. [file Image_1.tif]
